# Tetra­kis(pyridazine-κ*N*)bis­(thio­cyanato-κ*N*)nickel(II) pyridazine disolvate

**DOI:** 10.1107/S1600536812023306

**Published:** 2012-05-26

**Authors:** Susanne Wöhlert, Mario Wriedt, Inke Jess, Christian Näther

**Affiliations:** a Institut für Anorganische Chemie, Christian-Albrechts-Universität Kiel, Max-Eyth-Strasse 2, 24118 Kiel, Germany; b Departement of Chemistry, Texas A&M University, College Station, Texas 77843, USA

## Abstract

The reaction of nickel(II) thio­cyanate with an excess of pyridazine leads to single crystals of the title compound, [Ni(NCS)_2_(C_4_H_4_N_2_)_4_]·2C_4_H_4_N_2_. The Ni^II^ cations are coordinated by two terminal *N*-bonded thio­cyanate anions (*trans*) and four pyridazine ligands in a slightly distorted octa­hedral geometry. The discrete complexes are arranged into layers parallel to the *ab* plane which are separated by additional non-coordinated pyridazine ligands.

## Related literature

For related pyridazine coordination compounds, see: Boeckmann *et al.* (2011 ▶); Lloret *et al.* (1998 ▶); Yi *et al.* (2006 ▶); Wriedt & Näther (2009 ▶, 2011 ▶). 
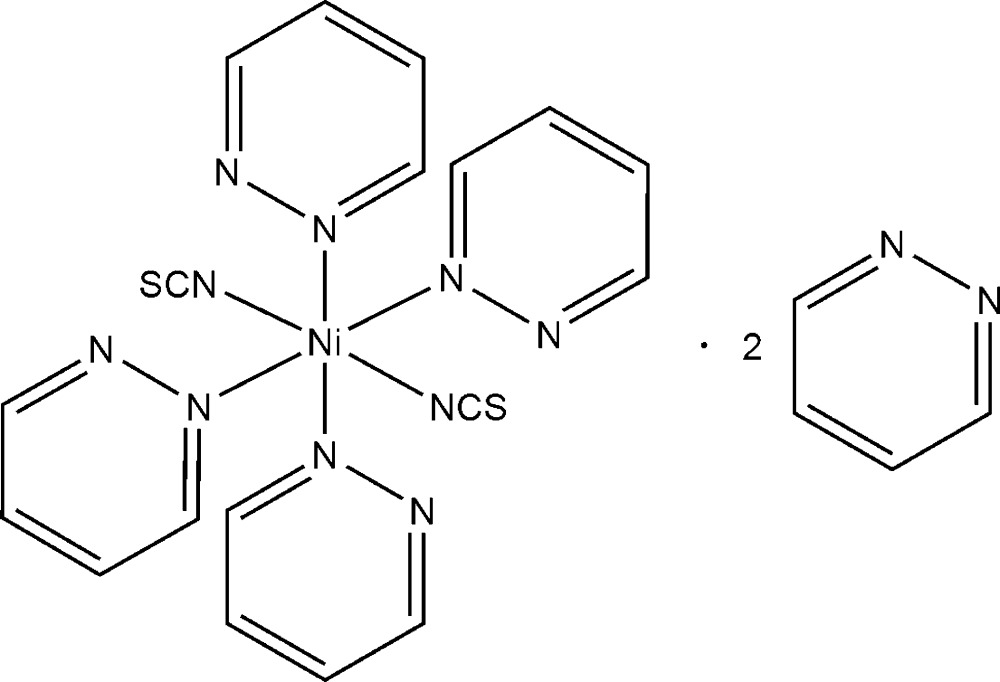


## Experimental

### 

#### Crystal data

[Ni(NCS)_2_(C_4_H_4_N_2_)_4_]·2C_4_H_4_N_2_
*M* *_r_* = 655.42
Triclinic, 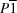

*a* = 11.2111 (9) Å
*b* = 12.033 (1) Å
*c* = 12.5409 (10) Å
α = 62.287 (9)°
β = 88.983 (10)°
γ = 88.949 (10)°
*V* = 1497.4 (2) Å^3^
*Z* = 2
Mo *K*α radiation
μ = 0.83 mm^−1^
*T* = 200 K
0.06 × 0.04 × 0.03 mm

#### Data collection

Stoe IPDS-1 diffractometer
Absorption correction: numerical (*X-SHAPE* and *X-RED32*; Stoe & Cie, 2008 ▶) *T*_min_ = 0.916, *T*_max_ = 0.973
11937 measured reflections
6400 independent reflections
4719 reflections with *I* > 2σ(*I*)
*R*_int_ = 0.029

#### Refinement

*R*[*F* ^2^ > 2σ(*F* ^2^)] = 0.032
*wR*(*F* ^2^) = 0.084
*S* = 0.97
6400 reflections
389 parameters
H-atom parameters constrained
Δρ_max_ = 0.39 e Å^−3^
Δρ_min_ = −0.40 e Å^−3^

### 

Data collection: *X-AREA* (Stoe & Cie, 2008 ▶); cell refinement: *X-AREA*; data reduction: *X-AREA*; program(s) used to solve structure: *SHELXS97* (Sheldrick, 2008 ▶); program(s) used to refine structure: *SHELXL97* (Sheldrick, 2008 ▶); molecular graphics: *XP* in *SHELXTL* (Sheldrick, 2008 ▶) and *DIAMOND* (Brandenburg, 2011 ▶); software used to prepare material for publication: *XCIF* in *SHELXTL* and *PLATON* (Spek, 2009 ▶).

## Supplementary Material

Crystal structure: contains datablock(s) I, global. DOI: 10.1107/S1600536812023306/bt5930sup1.cif

Structure factors: contains datablock(s) I. DOI: 10.1107/S1600536812023306/bt5930Isup2.hkl

Additional supplementary materials:  crystallographic information; 3D view; checkCIF report

## Figures and Tables

**Fig. 1.**
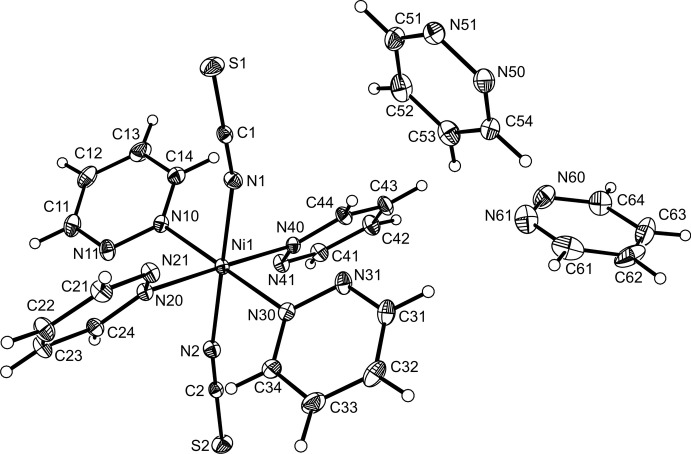
: Crystal structure of the title compound with atom labels and displacement ellipsoids drawn at the 50% probability level.

**Fig. 2.**
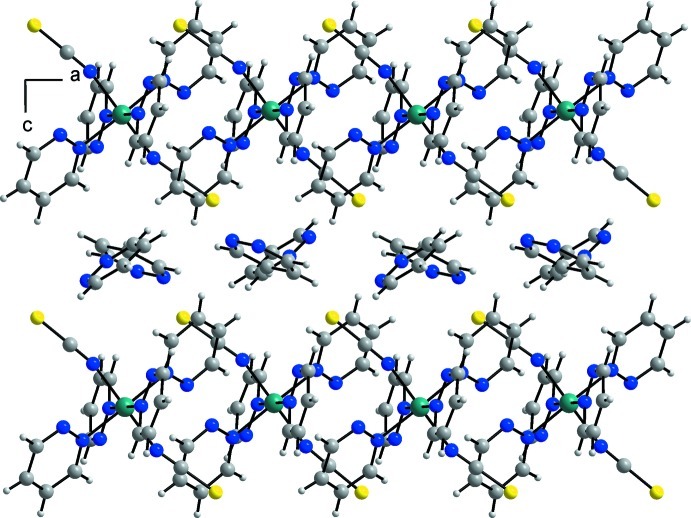
: Crystal structure of the title compound with view along the crystallographic b-axis.

## supplementary crystallographic information

### Comment

Currently, we are interested in the synthesis and characterization of coordination polymers based on Mn(II), Fe(II), Co(II), Ni(II) and Cd(II) thiocyanates and pyridazine as co-ligand. Only a few compounds based on Cobalt, Nickel and Cadmium are structurally characterized (Boeckmann *et al.*, 2011; Lloret *et al.*, 1998; Yi *et al.*, 2006; Wriedt & Näther, 2011; Wriedt & Näther, 2009). In this context we have reported on two different modifications of a trinuclear nickel(II) complex of composition [Ni_3_(NCS)_6_(pyridazine)_6_] (Wriedt & Näther, 2009). In our ongoing investigation in this field we have isolated light-green single-crystals of a further compound by the reaction of nickel(II) thiocyanate with an excess of pyridazine, that were characterized by single-crystal X-ray diffraction. In the crystal structure of the title compound each nickel(II) cation is coordinated by two terminal N-bonded thiocyanato anions and four pyridazine ligands in a slightly distorted octahedral geometry (Fig. 1). The NiN_6_ distances are ranges from 2.0494 (15) to 2.1530 (15) Å and the angles are between 87.33 (5) ° and 179.71 (7) °. Because of sterical reasons only one of the two pyridazine nitrogen atoms is involved in metal coordination. In the crystal structure the discrete complexes are arranged in layers that are parallel to the *ab* plane. These layers are separated by additional pyridazine ligands that are not coordinated to the metal centers (Fig. 2). The shortest intermolecular Ni···Ni distances amounts to 8.0823 (9) Å.

### Experimental

Nickel(II) thiocyanate (Ni(NCS)_2_) and pyridazine were obtained from Alfa Aesar. All chemicals were used without further purification. 0.125 mmol (21.7 mg) Ni(NCS)_2_ and 2.76 mmol (200 µ*L*) pyridazine were reacted in a closed snap-vial without stirring. Light-green single crystals of the title compounds were obtained after two weeks.

### Refinement

All H atoms were located in a difference map but were positioned with idealized geometry and were refined using a riding model with *U*_iso_(H) = 1.2 *U*_eq_(C) and C—H = 0.95 Å. *PLATON* (Spek, 2009) detected a pseudo-C centring in the structure. Nevertheless, the structure is just triclinic primitive.

### Crystal data

**Table:**

[Ni(NCS)_2_(C_4_H_4_N_2_)_4_]·2C_4_H_4_N_2_	*Z* = 2
*M**_r_* = 655.42	*F*(000) = 676
Triclinic, *P*1	*D*_x_ = 1.454 Mg m^−^^3^
Hall symbol: -P 1	Mo *K*α radiation, λ = 0.71073 Å
*a* = 11.2111 (9) Å	Cell parameters from 11937 reflections
*b* = 12.033 (1) Å	θ = 2.6–27.0°
*c* = 12.5409 (10) Å	µ = 0.83 mm^−^^1^
α = 62.287 (9)°	*T* = 200 K
β = 88.983 (10)°	Block, light-green
γ = 88.949 (10)°	0.06 × 0.04 × 0.03 mm
*V* = 1497.4 (2) Å^3^	

### Data collection

**Table:**

Stoe IPDS-1 diffractometer	6400 independent reflections
Radiation source: fine-focus sealed tube	4719 reflections with *I* > 2σ(*I*)
Graphite monochromator	*R*_int_ = 0.029
phi scan	θ_max_ = 27.0°, θ_min_ = 2.6°
Absorption correction: numerical (*X-SHAPE* and *X-RED32*; Stoe & Cie, 2008)	*h* = −14→14
*T*_min_ = 0.916, *T*_max_ = 0.973	*k* = −15→15
11937 measured reflections	*l* = −16→16

### Refinement

**Table:**

Refinement on *F*^2^	Secondary atom site location: difference Fourier map
Least-squares matrix: full	Hydrogen site location: inferred from neighbouring sites
*R*[*F*^2^ > 2σ(*F*^2^)] = 0.032	H-atom parameters constrained
*wR*(*F*^2^) = 0.084	*w* = 1/[σ^2^(*F*_o_^2^) + (0.0504*P*)^2^] where *P* = (*F*_o_^2^ + 2*F*_c_^2^)/3
*S* = 0.97	(Δ/σ)_max_ = 0.001
6400 reflections	Δρ_max_ = 0.39 e Å^−^^3^
389 parameters	Δρ_min_ = −0.40 e Å^−^^3^
0 restraints	Extinction correction: *SHELXL97* (Sheldrick, 2008), Fc^*^=kFc[1+0.001xFc^2^λ^3^/sin(2θ)]^-1/4^
Primary atom site location: structure-invariant direct methods	Extinction coefficient: 0.0149 (15)

### Special details

**Table:**

Geometry. All e.s.d.'s (except the e.s.d. in the dihedral angle between two l.s. planes) are estimated using the full covariance matrix. The cell e.s.d.'s are taken into account individually in the estimation of e.s.d.'s in distances, angles and torsion angles; correlations between e.s.d.'s in cell parameters are only used when they are defined by crystal symmetry. An approximate (isotropic) treatment of cell e.s.d.'s is used for estimating e.s.d.'s involving l.s. planes.
Refinement. Refinement of *F*^2^ against ALL reflections. The weighted *R*-factor *wR* and goodness of fit *S* are based on *F*^2^, conventional *R*-factors *R* are based on *F*, with *F* set to zero for negative *F*^2^. The threshold expression of *F*^2^ > σ(*F*^2^) is used only for calculating *R*-factors(gt) *etc*. and is not relevant to the choice of reflections for refinement. *R*-factors based on *F*^2^ are statistically about twice as large as those based on *F*, and *R*- factors based on ALL data will be even larger.

### Fractional atomic coordinates and isotropic or equivalent isotropic displacement parameters (Å^2^)

**Table:**

	*x*	*y*	*z*	*U*_iso_*/*U*_eq_	
Ni1	0.752765 (19)	0.75179 (2)	0.993234 (18)	0.01640 (8)	
N2	0.85894 (13)	0.65700 (14)	1.14111 (13)	0.0237 (3)	
C2	0.93634 (15)	0.60587 (16)	1.20694 (14)	0.0195 (3)	
S2	1.04648 (4)	0.53396 (5)	1.29882 (4)	0.03462 (13)	
N1	0.64734 (13)	0.84717 (14)	0.84542 (13)	0.0241 (3)	
C1	0.56476 (16)	0.89404 (16)	0.78585 (14)	0.0204 (4)	
S1	0.44826 (5)	0.95934 (5)	0.70169 (5)	0.03734 (14)	
N10	0.69744 (12)	0.57958 (14)	0.99727 (12)	0.0212 (3)	
N11	0.65668 (14)	0.48848 (14)	1.10237 (14)	0.0281 (3)	
C11	0.62296 (18)	0.38099 (18)	1.10720 (19)	0.0332 (4)	
H11	0.5923	0.3180	1.1815	0.040*	
C12	0.62968 (18)	0.3551 (2)	1.0107 (2)	0.0365 (5)	
H12	0.6056	0.2765	1.0180	0.044*	
C13	0.67269 (19)	0.4479 (2)	0.9041 (2)	0.0398 (5)	
H13	0.6806	0.4359	0.8346	0.048*	
C14	0.70445 (17)	0.5607 (2)	0.90134 (17)	0.0301 (4)	
H14	0.7323	0.6269	0.8275	0.036*	
N20	0.61071 (12)	0.74611 (13)	1.11065 (12)	0.0185 (3)	
N21	0.54160 (13)	0.85064 (14)	1.06923 (12)	0.0221 (3)	
C21	0.45590 (16)	0.85656 (18)	1.14045 (16)	0.0267 (4)	
H21	0.4084	0.9309	1.1115	0.032*	
C22	0.43142 (18)	0.7605 (2)	1.25495 (17)	0.0329 (5)	
H22	0.3682	0.7680	1.3027	0.039*	
C23	0.50158 (17)	0.65455 (18)	1.29662 (16)	0.0287 (4)	
H23	0.4887	0.5855	1.3740	0.034*	
C24	0.59298 (16)	0.65231 (16)	1.22036 (14)	0.0223 (4)	
H24	0.6444	0.5809	1.2481	0.027*	
N30	0.80605 (12)	0.92471 (14)	0.98921 (13)	0.0210 (3)	
N31	0.83861 (14)	1.02153 (14)	0.88415 (14)	0.0278 (3)	
C31	0.86974 (18)	1.12832 (19)	0.88297 (19)	0.0336 (4)	
H31	0.8940	1.1960	0.8084	0.040*	
C32	0.86879 (19)	1.1465 (2)	0.9847 (2)	0.0382 (5)	
H32	0.8915	1.2242	0.9804	0.046*	
C33	0.83376 (19)	1.0480 (2)	1.0911 (2)	0.0387 (5)	
H33	0.8298	1.0549	1.1635	0.046*	
C34	0.80415 (17)	0.93705 (19)	1.08911 (17)	0.0290 (4)	
H34	0.7815	0.8669	1.1627	0.035*	
N40	0.89770 (12)	0.75517 (13)	0.87968 (12)	0.0192 (3)	
N41	0.96253 (13)	0.64770 (14)	0.92420 (12)	0.0228 (3)	
C41	1.05424 (16)	0.64035 (18)	0.85962 (16)	0.0266 (4)	
H41	1.0987	0.5640	0.8902	0.032*	
C42	1.08890 (17)	0.7384 (2)	0.74940 (17)	0.0316 (4)	
H42	1.1567	0.7303	0.7069	0.038*	
C43	1.02254 (18)	0.84661 (19)	0.70416 (16)	0.0298 (4)	
H43	1.0420	0.9163	0.6291	0.036*	
C44	0.92462 (16)	0.85029 (17)	0.77327 (15)	0.0236 (4)	
H44	0.8756	0.9236	0.7429	0.028*	
N50	0.70575 (16)	1.39686 (16)	0.45667 (15)	0.0364 (4)	
N51	0.61715 (16)	1.33478 (17)	0.43752 (15)	0.0374 (4)	
C51	0.6319 (2)	1.2132 (2)	0.47219 (19)	0.0387 (5)	
H51	0.5686	1.1701	0.4587	0.046*	
C52	0.7336 (2)	1.1454 (2)	0.5268 (2)	0.0403 (5)	
H52	0.7403	1.0583	0.5501	0.048*	
C53	0.82377 (19)	1.2079 (2)	0.54582 (19)	0.0371 (5)	
H53	0.8959	1.1666	0.5831	0.044*	
C54	0.80526 (19)	1.3355 (2)	0.50801 (18)	0.0339 (5)	
H54	0.8675	1.3812	0.5197	0.041*	
N60	1.18977 (17)	1.12073 (18)	0.50816 (18)	0.0425 (4)	
N61	1.12255 (18)	1.1575 (2)	0.57472 (19)	0.0497 (5)	
C61	1.1514 (3)	1.2601 (3)	0.5806 (2)	0.0525 (7)	
H61	1.1019	1.2854	0.6279	0.063*	
C62	1.2485 (3)	1.3331 (2)	0.5228 (3)	0.0559 (7)	
H62	1.2665	1.4061	0.5301	0.067*	
C63	1.3172 (2)	1.2955 (2)	0.4547 (3)	0.0535 (7)	
H63	1.3855	1.3411	0.4120	0.064*	
C64	1.2832 (2)	1.1884 (2)	0.4506 (2)	0.0444 (6)	
H64	1.3301	1.1613	0.4030	0.053*	

### Atomic displacement parameters (Å^2^)

**Table:**

	*U*^11^	*U*^22^	*U*^33^	*U*^12^	*U*^13^	*U*^23^
Ni1	0.01669 (12)	0.01298 (12)	0.01687 (12)	0.00196 (7)	0.00033 (7)	−0.00480 (8)
N2	0.0236 (7)	0.0211 (8)	0.0227 (7)	0.0033 (6)	−0.0016 (6)	−0.0072 (6)
C2	0.0225 (8)	0.0150 (8)	0.0199 (7)	−0.0005 (7)	0.0014 (7)	−0.0073 (7)
S2	0.0301 (3)	0.0309 (3)	0.0348 (3)	0.0052 (2)	−0.0147 (2)	−0.0082 (2)
N1	0.0249 (8)	0.0231 (8)	0.0215 (7)	0.0036 (6)	−0.0018 (6)	−0.0081 (6)
C1	0.0262 (9)	0.0146 (8)	0.0191 (7)	−0.0016 (7)	0.0019 (7)	−0.0068 (7)
S1	0.0335 (3)	0.0295 (3)	0.0393 (3)	0.0034 (2)	−0.0174 (2)	−0.0074 (2)
N10	0.0193 (7)	0.0183 (8)	0.0248 (7)	−0.0001 (6)	0.0008 (6)	−0.0090 (6)
N11	0.0330 (8)	0.0186 (8)	0.0301 (8)	−0.0047 (7)	0.0069 (7)	−0.0091 (7)
C11	0.0324 (10)	0.0206 (10)	0.0431 (11)	−0.0054 (8)	0.0038 (9)	−0.0118 (9)
C12	0.0315 (10)	0.0283 (11)	0.0568 (13)	−0.0053 (8)	−0.0018 (9)	−0.0256 (10)
C13	0.0416 (12)	0.0464 (14)	0.0484 (12)	−0.0086 (10)	−0.0005 (10)	−0.0361 (11)
C14	0.0308 (10)	0.0336 (11)	0.0295 (9)	−0.0064 (8)	0.0011 (8)	−0.0175 (8)
N20	0.0192 (7)	0.0154 (7)	0.0195 (6)	0.0027 (6)	0.0004 (5)	−0.0070 (6)
N21	0.0226 (7)	0.0183 (8)	0.0222 (7)	0.0053 (6)	0.0007 (6)	−0.0070 (6)
C21	0.0270 (9)	0.0252 (10)	0.0286 (9)	0.0076 (8)	0.0008 (7)	−0.0136 (8)
C22	0.0325 (10)	0.0377 (12)	0.0293 (9)	0.0027 (9)	0.0093 (8)	−0.0168 (9)
C23	0.0337 (10)	0.0272 (10)	0.0204 (8)	0.0000 (8)	0.0055 (7)	−0.0071 (8)
C24	0.0260 (9)	0.0181 (9)	0.0203 (8)	0.0013 (7)	0.0006 (7)	−0.0068 (7)
N30	0.0185 (7)	0.0172 (7)	0.0260 (7)	0.0019 (6)	−0.0016 (6)	−0.0089 (6)
N31	0.0319 (8)	0.0174 (8)	0.0314 (8)	−0.0033 (6)	0.0060 (7)	−0.0093 (7)
C31	0.0341 (10)	0.0224 (10)	0.0412 (11)	−0.0069 (8)	0.0062 (9)	−0.0123 (9)
C32	0.0348 (11)	0.0295 (11)	0.0588 (13)	−0.0059 (9)	−0.0007 (10)	−0.0275 (11)
C33	0.0408 (12)	0.0428 (13)	0.0445 (11)	−0.0090 (10)	−0.0011 (9)	−0.0302 (11)
C34	0.0325 (10)	0.0276 (10)	0.0281 (9)	−0.0054 (8)	−0.0010 (8)	−0.0138 (8)
N40	0.0194 (7)	0.0157 (7)	0.0205 (6)	0.0001 (6)	0.0011 (5)	−0.0068 (6)
N41	0.0234 (7)	0.0181 (8)	0.0240 (7)	0.0045 (6)	0.0023 (6)	−0.0075 (6)
C41	0.0260 (9)	0.0260 (10)	0.0278 (9)	0.0054 (8)	0.0025 (7)	−0.0127 (8)
C42	0.0292 (10)	0.0374 (12)	0.0289 (9)	0.0002 (8)	0.0104 (8)	−0.0164 (9)
C43	0.0363 (10)	0.0280 (10)	0.0205 (8)	−0.0062 (8)	0.0080 (7)	−0.0076 (8)
C44	0.0311 (9)	0.0159 (9)	0.0200 (8)	0.0015 (7)	0.0013 (7)	−0.0053 (7)
N50	0.0455 (10)	0.0223 (9)	0.0359 (9)	−0.0048 (8)	−0.0030 (8)	−0.0086 (7)
N51	0.0394 (10)	0.0320 (10)	0.0333 (9)	−0.0052 (8)	−0.0048 (7)	−0.0086 (8)
C51	0.0455 (13)	0.0367 (12)	0.0370 (10)	−0.0160 (10)	0.0042 (9)	−0.0192 (10)
C52	0.0516 (14)	0.0242 (11)	0.0462 (12)	−0.0015 (10)	0.0107 (10)	−0.0176 (10)
C53	0.0339 (11)	0.0377 (12)	0.0352 (10)	0.0051 (9)	0.0074 (8)	−0.0137 (9)
C54	0.0351 (11)	0.0321 (11)	0.0322 (10)	−0.0095 (9)	0.0004 (8)	−0.0127 (9)
N60	0.0403 (10)	0.0337 (10)	0.0583 (12)	−0.0004 (8)	−0.0048 (9)	−0.0254 (9)
N61	0.0437 (11)	0.0400 (12)	0.0573 (12)	0.0049 (9)	0.0056 (9)	−0.0163 (10)
C61	0.0640 (17)	0.0532 (16)	0.0474 (13)	0.0264 (14)	−0.0112 (12)	−0.0299 (13)
C62	0.0652 (17)	0.0297 (13)	0.0843 (19)	0.0167 (12)	−0.0445 (15)	−0.0353 (14)
C63	0.0351 (12)	0.0342 (13)	0.0771 (18)	−0.0056 (10)	−0.0074 (12)	−0.0136 (13)
C64	0.0445 (13)	0.0424 (14)	0.0494 (13)	0.0022 (11)	0.0042 (10)	−0.0242 (11)

### Geometric parameters (Å, º)

**Table:**

Ni1—N1	2.0494 (15)	C32—C33	1.366 (3)
Ni1—N2	2.0538 (15)	C32—H32	0.9500
Ni1—N40	2.1298 (13)	C33—C34	1.393 (3)
Ni1—N20	2.1299 (13)	C33—H33	0.9500
Ni1—N10	2.1516 (15)	C34—H34	0.9500
Ni1—N30	2.1530 (15)	N40—C44	1.327 (2)
N2—C2	1.160 (2)	N40—N41	1.3494 (19)
C2—S2	1.6387 (18)	N41—C41	1.325 (2)
N1—C1	1.161 (2)	C41—C42	1.391 (3)
C1—S1	1.6362 (18)	C41—H41	0.9500
N10—C14	1.325 (2)	C42—C43	1.365 (3)
N10—N11	1.342 (2)	C42—H42	0.9500
N11—C11	1.328 (3)	C43—C44	1.399 (2)
C11—C12	1.384 (3)	C43—H43	0.9500
C11—H11	0.9500	C44—H44	0.9500
C12—C13	1.369 (3)	N50—C54	1.326 (3)
C12—H12	0.9500	N50—N51	1.343 (3)
C13—C14	1.394 (3)	N51—C51	1.326 (3)
C13—H13	0.9500	C51—C52	1.382 (3)
C14—H14	0.9500	C51—H51	0.9500
N20—C24	1.327 (2)	C52—C53	1.360 (3)
N20—N21	1.3502 (19)	C52—H52	0.9500
N21—C21	1.324 (2)	C53—C54	1.393 (3)
C21—C22	1.388 (3)	C53—H53	0.9500
C21—H21	0.9500	C54—H54	0.9500
C22—C23	1.370 (3)	N60—C64	1.318 (3)
C22—H22	0.9500	N60—N61	1.331 (3)
C23—C24	1.398 (2)	N61—C61	1.316 (3)
C23—H23	0.9500	C61—C62	1.378 (4)
C24—H24	0.9500	C61—H61	0.9500
N30—C34	1.328 (2)	C62—C63	1.360 (4)
N30—N31	1.341 (2)	C62—H62	0.9500
N31—C31	1.331 (3)	C63—C64	1.375 (4)
C31—C32	1.391 (3)	C63—H63	0.9500
C31—H31	0.9500	C64—H64	0.9500
			
N1—Ni1—N2	179.71 (7)	N31—C31—H31	118.1
N1—Ni1—N40	90.25 (6)	C32—C31—H31	118.1
N2—Ni1—N40	89.64 (6)	C33—C32—C31	117.2 (2)
N1—Ni1—N20	91.20 (6)	C33—C32—H32	121.4
N2—Ni1—N20	88.90 (6)	C31—C32—H32	121.4
N40—Ni1—N20	178.54 (6)	C32—C33—C34	117.37 (19)
N1—Ni1—N10	88.37 (6)	C32—C33—H33	121.3
N2—Ni1—N10	91.90 (6)	C34—C33—H33	121.3
N40—Ni1—N10	87.95 (5)	N30—C34—C33	123.06 (18)
N20—Ni1—N10	92.16 (5)	N30—C34—H34	118.5
N1—Ni1—N30	91.25 (6)	C33—C34—H34	118.5
N2—Ni1—N30	88.48 (6)	C44—N40—N41	120.64 (14)
N40—Ni1—N30	92.57 (5)	C44—N40—Ni1	125.54 (12)
N20—Ni1—N30	87.33 (5)	N41—N40—Ni1	113.81 (10)
N10—Ni1—N30	179.36 (5)	C41—N41—N40	118.32 (14)
C2—N2—Ni1	165.91 (13)	N41—C41—C42	123.54 (17)
N2—C2—S2	179.46 (15)	N41—C41—H41	118.2
C1—N1—Ni1	161.54 (13)	C42—C41—H41	118.2
N1—C1—S1	179.63 (17)	C43—C42—C41	117.98 (16)
C14—N10—N11	119.91 (16)	C43—C42—H42	121.0
C14—N10—Ni1	122.41 (13)	C41—C42—H42	121.0
N11—N10—Ni1	117.67 (11)	C42—C43—C44	117.04 (16)
C11—N11—N10	118.70 (15)	C42—C43—H43	121.5
N11—C11—C12	124.07 (19)	C44—C43—H43	121.5
N11—C11—H11	118.0	N40—C44—C43	122.43 (16)
C12—C11—H11	118.0	N40—C44—H44	118.8
C13—C12—C11	116.88 (19)	C43—C44—H44	118.8
C13—C12—H12	121.6	C54—N50—N51	119.27 (18)
C11—C12—H12	121.6	C51—N51—N50	118.58 (19)
C12—C13—C14	117.59 (18)	N51—C51—C52	124.1 (2)
C12—C13—H13	121.2	N51—C51—H51	117.9
C14—C13—H13	121.2	C52—C51—H51	117.9
N10—C14—C13	122.82 (18)	C53—C52—C51	117.5 (2)
N10—C14—H14	118.6	C53—C52—H52	121.2
C13—C14—H14	118.6	C51—C52—H52	121.2
C24—N20—N21	120.47 (13)	C52—C53—C54	116.8 (2)
C24—N20—Ni1	124.29 (11)	C52—C53—H53	121.6
N21—N20—Ni1	115.09 (10)	C54—C53—H53	121.6
C21—N21—N20	118.35 (14)	N50—C54—C53	123.7 (2)
N21—C21—C22	123.81 (17)	N50—C54—H54	118.1
N21—C21—H21	118.1	C53—C54—H54	118.1
C22—C21—H21	118.1	C64—N60—N61	118.7 (2)
C23—C22—C21	117.67 (16)	C61—N61—N60	119.0 (2)
C23—C22—H22	121.2	N61—C61—C62	124.4 (2)
C21—C22—H22	121.2	N61—C61—H61	117.8
C22—C23—C24	117.13 (17)	C62—C61—H61	117.8
C22—C23—H23	121.4	C63—C62—C61	116.7 (2)
C24—C23—H23	121.4	C63—C62—H62	121.7
N20—C24—C23	122.52 (16)	C61—C62—H62	121.7
N20—C24—H24	118.7	C62—C63—C64	116.9 (2)
C23—C24—H24	118.7	C62—C63—H63	121.6
C34—N30—N31	119.93 (16)	C64—C63—H63	121.6
C34—N30—Ni1	120.60 (12)	N60—C64—C63	124.4 (2)
N31—N30—Ni1	119.45 (11)	N60—C64—H64	117.8
C31—N31—N30	118.68 (16)	C63—C64—H64	117.8
N31—C31—C32	123.75 (19)		
